# Anticancer Activity of Ether Derivatives of Chrysin

**DOI:** 10.3390/molecules30040960

**Published:** 2025-02-19

**Authors:** Arkadiusz Sokal, Patryk Mruczek, Mateusz Niedoba, Agnieszka Dewalska, Klaudia Stocerz, Monika Kadela-Tomanek

**Affiliations:** 1 Department of Organic Chemistry, Faculty of Pharmaceutical Sciences in Sosnowiec, Medical University of Silesia, 4 Jagiellońska Str., 41-200 Sosnowiec, Poland; asokal@outlook.com (A.S.); s84812@365.sum.edu.pl (P.M.); 2 Doctoral School, Medical University of Silesia in Katowice, 15 Poniatowskiego Str., 40-055 Katowice, Poland; d201280@365.sum.edu.pl (M.N.); d201303@365.sum.edu.pl (A.D.); kstocerz@outlook.com (K.S.); 3 Department of Pathology, Faculty of Pharmaceutical Sciences in Sosnowiec, Medical University of Silesia, 30 Ostrogórska Str., 41-200 Sosnowiec, Poland; 4 Department of Microbiology and Immunology, Faculty of Medical Sciences in Zabrze, Medical University of Silesia, 19 Jordana Str., 41-800 Zabrze, Poland; 5 Department of Community Pharmacy, Faculty of Pharmaceutical Sciences in Sosnowiec, Medical University of Silesia in Katowice, 10 Jedności Str., 41-200 Sosnowiec, Poland

**Keywords:** chrysin, chrysin derivatives, anticancer

## Abstract

Chrysin, a naturally occurring flavonoid, exhibits a broad spectrum of biological activities, including showing anticancer properties. However, its clinical application is limited by poor bioavailability and low solubility. The introduction of an amine, amide, ester, or alkoxy group to a flavone skeleton influences the biological activity. This review also discusses hybrid compounds, such as the chrysin–porphyrin hybrid, which are characterized by higher biological activity and better bioavailability properties than single molecules. This review concentrates on the anticancer activity of chrysin and its derivatives against the most popular cancers, such as breast, lung, prostate, and gastrointestinal tumors.

## 1. Introduction

According to an Organization for Economic Co-operation and Development (OECD) report, cancer was a cause of death of about 1 out of 5 people in OECD countries in 2021. This makes it the second leading cause of death after circulatory diseases [1,2]. One of the most important causes of the high mortality rate is the low effects of used anticancer treatment. For this reason, the research of novel cancer therapies is very important [3,4].

Substances of natural origin have a major role in the development of pharmaceutics. It is estimated that plants have been used in medicine for more than 65,000 years. However, the first described examples of the use of natural substances as medical preparations were found on Sumerian clay slabs dated before 5000 years ago [5,6]. Despite the progress in drug development, substances from natural sources are still important. It is estimated that more than 40% of current pharmaceutics are obtained from plants or are semi-synthetic derivatives of natural substances [7,8].

One of the important classes of natural substances exhibiting high biological activity is flavonoids, which occur in many plants, fruits, and mushrooms [9,10]. Flavonoids belong to a family of phenolic compounds and polyphenols that include more than 6000 different structures. The flavonoid scaffold consists of fifteen carbons, which create two phenyl rings and one pyran moiety [11,12].

**Figure 1:**
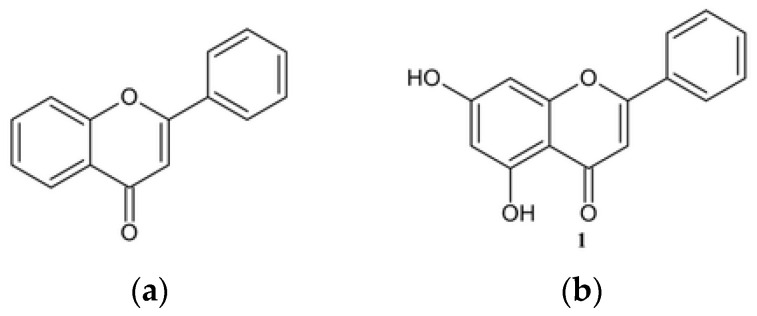
The chemical structure of a flavone (**a**) and chrysin (**b**).

One of the subgroups of flavonoids is flavones, which are widely distributed in the plant kingdom. Flavones exhibit high antioxidant activity [13,14,15], protect cell membranes [16,17], reduce lipid levels [16], and inhibit xanthan oxidase [18]. In addition, they exhibit many pharmaceutical effects, including, in particular, anticancer activity, which is related to the structure of flavones. Their basic structure consists of two benzene rings (A and B) linked by an oxygen-containing heterocyclic ring. The characteristic features of flavones include a carbonyl group attached to the carbon atom at the C4 position and a double bond between C2 and C3 (Figure 1a) [13,14,15,19].

The double bond at the pyran moiety and the carbonyl group at the C4 position influence the biological activity of flavones. According to the literature data, this fragment of the flavone structure is responsible for the anticancer activity of the compound [11,15]. Most widespread flavones are hydroxyflavones, which contain one or more hydroxyl groups. An example of hydroxylated flavone is chrysin **1**, which contains two hydroxyl groups at C5 and C7 positions (Figure 1b) [10,20]. The chemical modification of chrysin usually consists of the modification of two hydroxyl groups at the C5 and C7 positions. However, OH at the C7 position is more reactive and such derivatives are mostly described [21,22]. Compound **1** is found in many plant species, such as the passiflora (*Passiflora edulis Sims*), including its fruit, the bitter melon (*Momordica charantia*), walnut flowers (*Juglans regia*), and the wild Himalayan pear (*Pyrus pashia*) [23,24,25,26]. In addition, it is also found in honey, propolis, and even some mushroom species [25,27,28]. Chrysin **1** shows a broad spectrum of biological activity, such as anticancer [25,29,30,31,32,33], anti-inflammatory [34,35,36], antiasthmatic [34], antimicrobial [34], antiaging [37,38], antidiabetic [39], antidepressant [40,41], neuroprotective [42,43], cardioprotective [43,44,45,46], and hepatoprotective [47].

The use of compound **1** is limited due to low absorption after oral administration, which is equal to 5%. The low bioavailability of chrysin **1** causes poor water solubility and rapid degradation in the gastrointestinal tract [48,49]. The introduction of additional substituents influences the bioavailability and activity of semi-synthetic compounds. The search for derivatives of known compounds with proven biological activity is a critical aspect of modern drug discovery and development [50]. This review reports semi-synthetic and synthetic chrysin derivatives that exhibit anticancer activity published between 2014 and 2024.

## 2. Anticancer Activity and Bioavailability of Chrysin

The literature describes the anticancer effects of chrysin **1** on different types of cancer cell lines [30,33,51,52,53,54,55,56]. For example, compound **1** reduces the VEGF gene expression associated with induced hypoxia in breast cancer. This effect could reduce breast-to-lung metastasis and cell proliferation [53].

In prostate cancer cells, compound **1** causes downregulation of PI3K/Akt pathway expression, which is responsible for increased cell proliferation and metastasis formation [57,58,59,60]. Studies on cervical cancer cell lines show that chrysin **1** causes downregulation of the NF-κB pathway and increases caspase-3, caspase-9, and Bax levels, which can be indicated as leading to inhibition of the cell cycle between the G2/M phases and its apoptosis [56,61,62,63,64].

In gastrointestinal cancers, a particular role is played by a reduction in the activity of the enzyme ten-eleven translocation (TET), which interferes with tumor cell migration and causes apoptosis [52,65,66,67].

The literature describes that compound **1** increases the expression of peroxisome proliferator-activated receptor alpha (PARPα) [27,68]. PARPα induces the downregulation of CYP2S1 and CYP1B1 expression, which leads to tumor cycle arrest, the induction of apoptosis, and the reduction of tumor cell migration, inhibiting metastatic development [69].

Studies on the lung cancer cell line show that compound **1** reduces the risk of metastasis and decreases tumor cell viability [70].

The literature also describes the effect of chrysin **1** on microRNA expression. Compound **1** promotes the expression of miR-9 and Let-7a, tumor suppressor factors that inhibit cancer cell growth and proliferation [52,66].

Unfortunately, the use of chrysin **1** in cancer treatment is limited by its physical and chemical properties. One of the important problems is its poor water solubility, which limits absorption in the gastrointestinal tract [71]. Additionally, within the intestinal lumen, chrysin **1** can undergo biotransformation, such as oxidation, reduction, and condensation with other biomolecules, which can reduce the biological activity of the parent compound [72,73]. Furthermore, compound **1** is subject to intensive metabolism by the gut microbiota, which contributes to its degradation before systemic absorption occurs [74]. Chrysin **1** also interacts with membrane transporters, which may either facilitate or hinder its uptake, further complicating its pharmacokinetic profile [75]. Once absorbed, flavone **1** exhibits low plasma stability and a short half-life due to rapid hepatic metabolism via phase I and phase II enzymatic pathways [76,77]. This rapid metabolic transformation results in the elimination of chrysin through renal and fecal excretion, restricting its systemic availability.

Modern pharmaceutical formulations, such as nanocapsules [74], nano- and microemulsions [72], polymeric nanoparticles [71,73,75,78,79,80,81], liposomes [74,79], or micelles [71,73,78] allow us to obtain new forms of drugs, which could be the solution to the pharmacokinetic limitations of chrysin **1**. Another promising approach is the inclusion of excipients such as piperine, which can enhance chrysin **1** absorption by inhibiting metabolic enzymes responsible for its rapid degradation and modulating membrane transporter activity to improve cellular uptake [72]. Additionally, compounds acting as either inhibitors or activators of membrane transporters may further optimize chrysin’s pharmacokinetics by regulating its intestinal absorption and systemic distribution [72,74,75]. An alternative strategy is the synthesis of semi-synthetic and synthetic derivatives. Introduction of hydrophobic groups at the C5 and/or C7 position of chrysin affects stability against oxidative degradation, increases lipophilicity, and improves membrane permeability and bioavailability. Moreover, lipophilic moieties further increase solubility, reduce metabolism, prolong half-life, and increase effectiveness by preventing chemical and metabolic hydrolysis [72]. The structural modification does not only increase the bioavailability of chrysin derivatives but may also expand on biological activities. For example, a methylglyoxal derivative of chrysin exhibited enhanced anti-glycation activity and improved water solubility, demonstrating the potential of chemical modifications to optimize its pharmacological properties [82].

## 3. Anticancer Activity of Ether Derivatives of Chrysin

The anticancer activity of chrysin derivatives is a promising area of research in the search for new candidates for anticancer drugs in the natural environment. Numerous studies have shown great anticancer potential of chrysin **1**, but its derivatives, of both natural and synthetic origin, are the most promising candidates [20,29,30,33,40,83,84,85].

**Figure 2:**
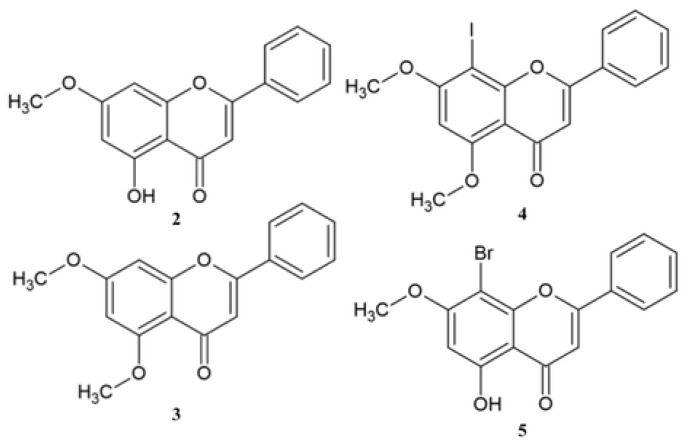
The chemical structure of methyl derivatives of chrysin **2**–**5**.

Methylated derivatives **2**–**5** are a well-studied group of compounds, which makes it possible to compare their anticancer activity on various cancer cell lines (Figure 2) [86,87,88,89]. According to the literature data, the methyl group influences the biochemical pathways. Cai et al. show that methylene derivatives of flavone reduce the COX and PEG-2 concentration, increasing anticancer activity compared with unmethylated derivatives [90].

Park et al., from *Apinia oxyphalla*, isolated tectochrysin **2** and examined its anticancer activity against human colon cancer cell lines (SW480 and HCT116), as well as in vivo xenograft–bearing nude mice bearing HCT116. The result shows that compound **2** inhibited both colon cancer cell lines in a concentration-dependent manner. Comparing the IC_50_ values shows that derivative **2** exhibited almost 1.5-times higher activity against the SW480 cell line than HCT116 (Table 1). Moreover, derivative **2** was not cytotoxic against normal CCD-18co cells. The in vivo studies prove that tumor growth was inhibited in 48.1%. The in silico studies show that compound **2** is well absorbed in the digestive tract [86]. Suradej et al. identified **2** as one of the active compounds of *Kaempferia parviflora* extract, which was proven to inhibit STAT3 activation as well as the production of IL-6 in HeLa cervical cancer cells [87].

Bae et al. obtained 5,7-dimethoxyflavone **3** and tested its anticancer activity alone and in a mixture of 10 μM compound **3** with (−)-epigallocatechin-3-*O*-gallate (EGCG) (Table 2). As seen in Table 2, the combination of **3** with EGCG increases the activity against the tested cell line [88]. Walle et al. showed that derivative **3** inhibits synthesis of DNA in tongue (SCC-9) cell line, and the effect was 10-times higher compared with chrysin **1** [91].

Compounds **4**–**5** were synthesized by Zheng et al., and their anticancer activity was tested in vitro against SGC-7901 and HT-29 cell lines. Derivative **4** exhibits comparable activity against both cell lines, whereas derivative 5 shows higher activity against HT-29 (Table 3). Comparing the activity of derivatives **4**–**5** with chrysin **1** shows that they had better activity than natural substance **1** [89].

Chrysin derivatives with ether groups have gained significant attention due to their diverse biological activities. Numerous studies have demonstrated that these compounds exhibit remarkable anticancer, antibacterial, and antifungal properties [92,93,94,95,96,97,98].

**Figure 3:**
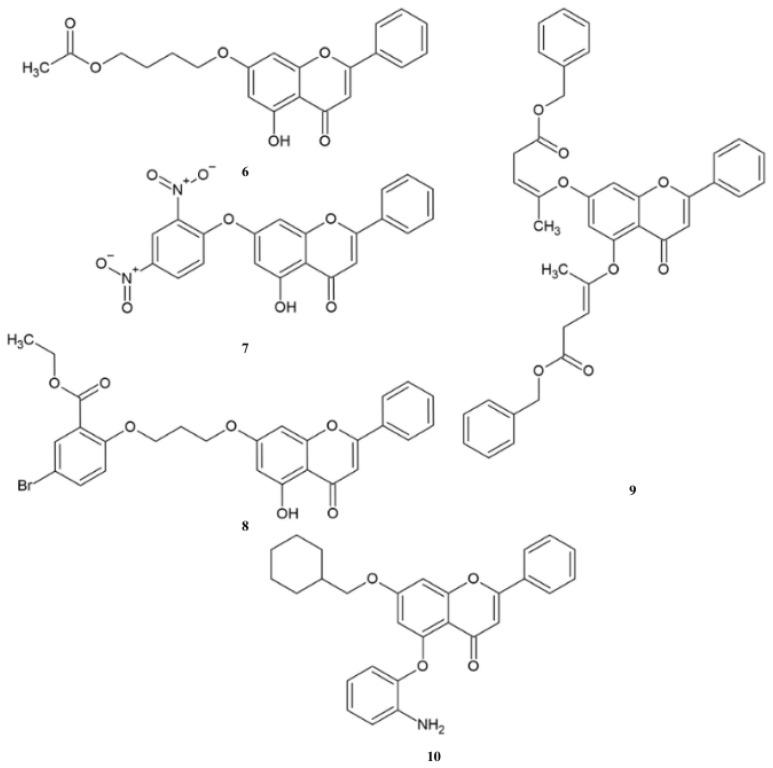
The chemical structure of ether compound **6**–**10**.

Omonga et al. synthesized derivatives **6**–**7** and tested their anticity against a panel of human cancer cell lines (Figure 3) [99].

Anticancer activity of compounds **6**–**7** against tested cell lines is in the range of 1.56 μM to 33.5 μM (Table 4). Both compounds exhibit the highest activity against colon cancer HCT116. Comparing the IC_50_ values against tested cell lines shows that the most important difference is observed for the HepG2 line. For this cell line, compound **7** exhibits almost 4-times higher activity than **6.** The cytotoxicity of derivatives was determined against the Beas-2B cell line. The tested compounds showed low toxicity and obtained IC_50_, which was more than 100 μM [99].

Compound **8** was tested in vivo in a mouse model, as well as in vitro against the MCF-7, HepG2, MGC-803, and MFC cell lines (Table 5). Derivative **8** exhibited low anticancer activity against the tested cell lines. Comparison of the IC_50_ shows that flavone **8** is characterized by better activity against MGC-803 and MFC cells. Moreover, the in vivo test showed that in mice treated with **8** (40 mg/kg), the tumor volume at the end of the experiment was almost 4-times smaller than in the group treated with golden standard 5-Fu, without the impact on total body weight in comparison to the 5-Fu group [100].

Jin et al. synthesized compound **9**, which was tested in vitro against K562, A549, HEL, and PC3. The activity was measured as a percentage of inhibition rate (Table 6). The highest activity was observed against the HEL cell line, which was almost 5- and 10-times more active than the K562 and A549 cell lines, respectively. Moreover, the compound did not inhibit the growth of the PC3 cell line [101].

Tang et al. performed synthesis of derivative **10** and analyzed in vitro its anticancer activity against Hela, BGC823, MCF-7, HepG2, and normal cells HEK-293 [102]. The results show that compound **10** inhibited the growth of the BGC823, MCF7, and HepG2 cell lines. Moreover, derivative **10** in the concentration 62.5 μM causes a 60% survival rate in normal cells HEK-293. Ether **10** at concentrations of 2.5–10 μM markedly increased the cytotoxic effects of 10-hydroxy camptothecin in cancer cells, with a particularly pronounced impact on gastric cancer BGC823 cells and breast cancer MCF7 cells.

**Figure 4:**
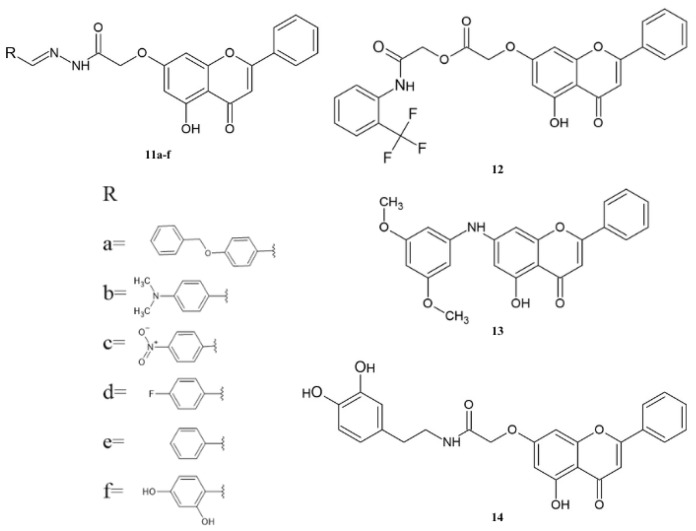
The chemical structure of compounds **11**–**14**.

Interesting derivatives of chrysin are amine [103,104,105], imine [106,107,108,109,110,111], and, in particular, amide [110,111,112,113,114,115,116,117] derivatives that show significant anticancer activity (Figure 4).

Al-Oudat et al. synthesized and tested the anticancer activity of derivatives **11a**–**f** against breast cancer cell lines (MDA-MB-231 and MCF-7). As a reference substance, they used doxorubicin. The anticancer activity of derivatives **11a**–**f** are presented in Table 7 [118,119].

The tested compounds are characterized by lower activity against the tested cell lines than doxorubicin. Comparing the activity of **11a**–**d** shows that the compound exhibits higher activity than the MDA-MB-231 cell line than MCF-7. Comparing the IC_50_ value of derivatives **11e** and **11f** against MDA-MB-231 shows that the introduction of a hydroxyl group at the C3 position of the phenyl substituent increases the activity. Compounds **11e**–**f** were also tested against breast cancer (BT-20), brain cancer (U-251), and colon cancer (HCT116 and HMEC). The results showed that derivatives **11f** had higher activity than **6e**, and the obtained IC_50_ values were in the range of 2.68 μM to 8 μM and 10.43 μM to 100 μM, respectively.

The anticancer activities of compounds **12** and **13** were tested against lung (A549), colon (HCT-116), central nervous system (U251), and ovarian (OVCAR-3) cancer cell lines (Table 8). Derivative **12** exhibited moderate activity against the tested cell lines, and the IC_50_ is equal from 7.99 μM to 8.99 μM. Meanwhile, compound **13** shows low activity against the tested cell lines, except for HCT-15, for which the IC_50_ is equal to 0.06 μM [55].

The amide derivative **14** was tested against breast cancer cell lines (Table 9). The results show that compound **14** has higher activity against all tested cell lines than reference substance **1**. Flavone **14** shows high activity against triple-negative breast cancer cell lines (MDA-MB-231 and MDA-MB-453). Moreover, amide **14** does not influence the normal cell line HEK293 (IC_50_ = 53.04 μM) [120].

**Figure 5:**
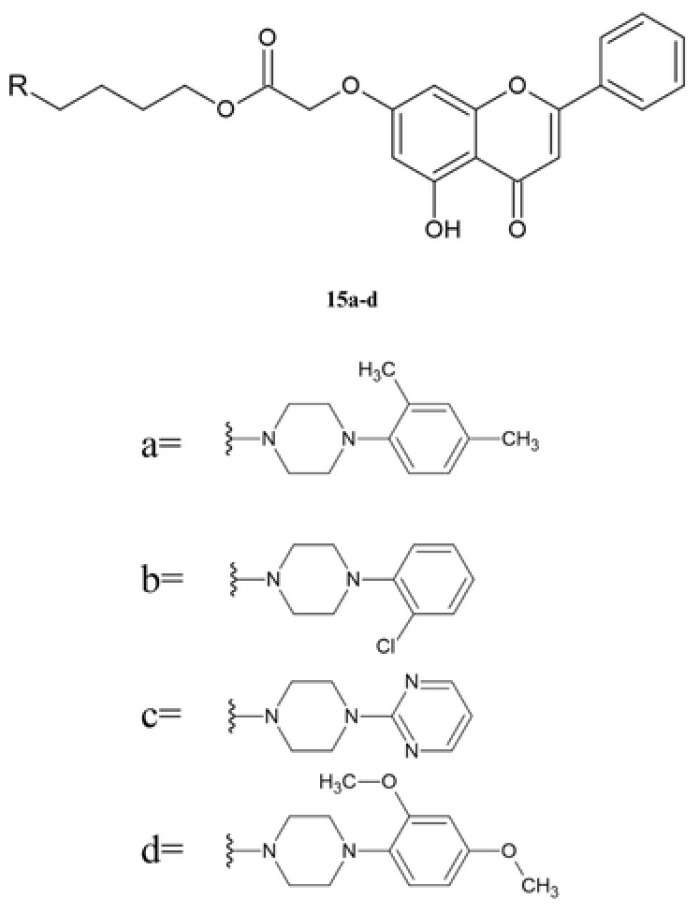
The chemical structure of derivatives **15a**–**d**.

Patel et al. obtained piperazine derivatives **15a**–**d** in the multistep reaction (Figure 5) [32].

The obtained compounds **15a**–**d** were tested in vitro against cervical cancer cell lines (HeLa, CaSki) and an ovarian cancer cell line (SK-OV-3) using SRB assay. The toxicity of the compounds was evaluated using the Madin-Darby canine kidney (MDCK) cell line (Table 10).

In the series of compounds **15a**–**d**, the highest activity against the HeLa cell line is shown by derivative **15c**, containing pyrimidine moiety. However, this compound is characterized by the highest toxicity against the normal cell line (MDCK). The presence and position of electron-donating and electron-withdrawing groups on the piperazine moiety played a significant role in enhancing its anticancer activity. Compounds **15a**, **15b**, and **15d** contain differently substituted phenyl rings. The introduction of a chloride atom (**15b**) increases the activity against the CaSki line compared to derivatives **15a**–**b**. Moreover, derivative **15b** shows low toxicity against the MDCK line. Comparing the compounds with methyl group (**15a**) and methoxy group (**15d**) shows that ether group reduces the activity against the HeLa and CaSki cell lines. Compound **15a** is characterized by a high selectivity index against all tested cell lines [32].

**Figure 6:**
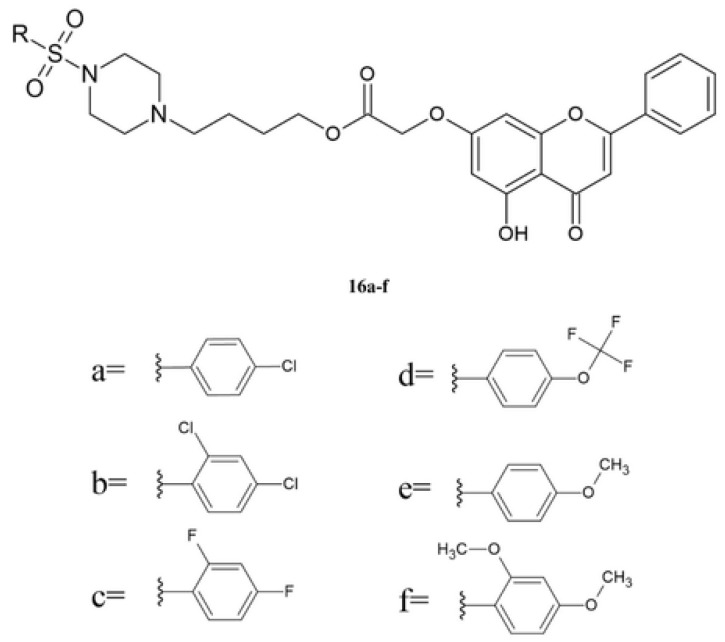
The chemical structure of derivatives **16a**–**f**.

Patel et al. synthesized six various chrysin sulfonylpiperazine derivatives **16a**–**f** (Figure 6) [121].

Compounds **16a**–**f** were tested against four human cancer cell lines (SK-OV-3, HeLa, A549, and HT-29) and normal kidney tissue (MDCK). As a reference substance, gefitinib was used (Table 11) [121].

Derivative **16c**, which contains two fluorine atom substitutions on the sulfonylpiperazine nucleus, exhibited the highest activity against the SK-OV-3 cell line, and the activity is similar to Gefitinib, the reference substance. Comparing the activity of **16b** and **16c** shows that the type of halogen atom influences the anticancer effect. Compound **16e** contains the methoxy group at the C4 position of the phenyl ring and exhibits lower activity against SK-OV-3 compared to derivative **16f**, which contains two methoxy groups at the C2 and C4 positions of the phenyl ring (Table 3). The introduction of a trifluoromethoxy group (**16d**) reduces the activity against the SK-OV-3 line compared with compound **16e**. Comparing the activity against the HeLa cell line of derivatives **16a**–**c** shows that it is in the order of **16c** > **16b** > **16a**, which means that compounds with two halogen atoms had high activity against this cell line. The tested compounds **16a**–**f** showed low activity against the A549 and HT-29 cell lines, and the obtained IC_50_ values are lower than that obtained for the reference substance (Table 3). The synthesis compounds were characterized by low toxicity against the normal cell line (MDCK), and the IC_50_ is higher than 200 μg/mL

The analysis of the structure–activity relationship shows that for compounds **16a**–**f**, there exists a strong correlation between the substituent in the phenyl moiety and the biological effect. Compounds **16a**–**c**, containing electron-withdrawing halogen substituents, such as chlorine and fluorine, exhibit higher activity against cancer cell lines compared to those with electron-donating groups. In this group of compounds, the derivative **16c** shows the highest activity, which may be caused by the presence of two strong electron-withdrawing substituents. The introduction of an electronegative group influences the physicochemical properties, such as lipophilicity and metabolic stability. Meanwhile, compounds **16d**–**f**, which contain electron-donating groups, showed lower anticancer activity. The tested compounds **16**–**f** showed low toxicity, and IC_50_ values against MDCK cells are in the range of 163.6 µg/mL to 296.8 µg/mL [121].

**Figure 7:**
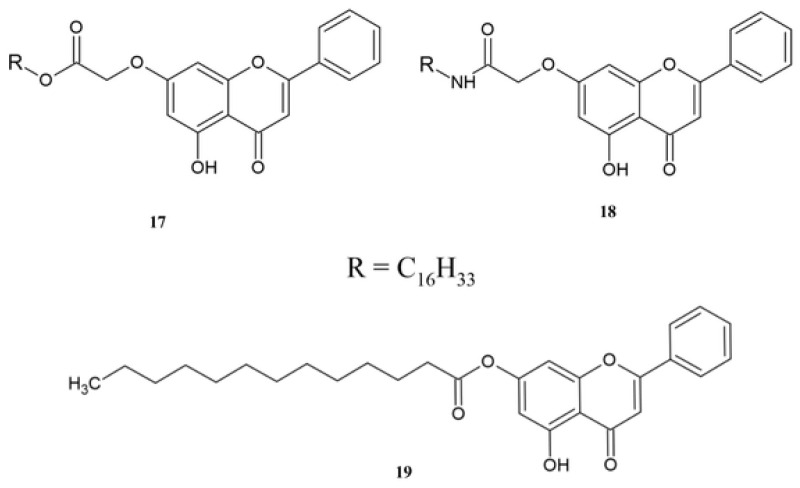
The chemical structure of derivatives **17**–**19**.

The literature describes the amide and ester derivatives of chrysin **17**–**19**, which were tested as an anticancer target against the HT-29 cell line and epidermal growth factor receptor kinase (EGFR) (Figure 7) [122].

As seen in Table 12, the amide derivative **18** has 2-times higher activity against the HT-29 cell line than compound **17**. Both compounds cause the inhibition of the EGFR receptor. However, the replacement of an amide group with an ester group slightly reduces the activity on the EGFR receptor (Table 12) [122].

Chen et al. described the synthesis and anticancer activity of ester **19** [123]. The introduction of a chain with twenty carbon atoms at the C7 position of chrysin increases activity against the HepG2 cell line compared to chrysin **1**, and the IC_50_ is 5-times higher for derivative **19** than that obtained for compound **1** [123].

**Figure 8:**
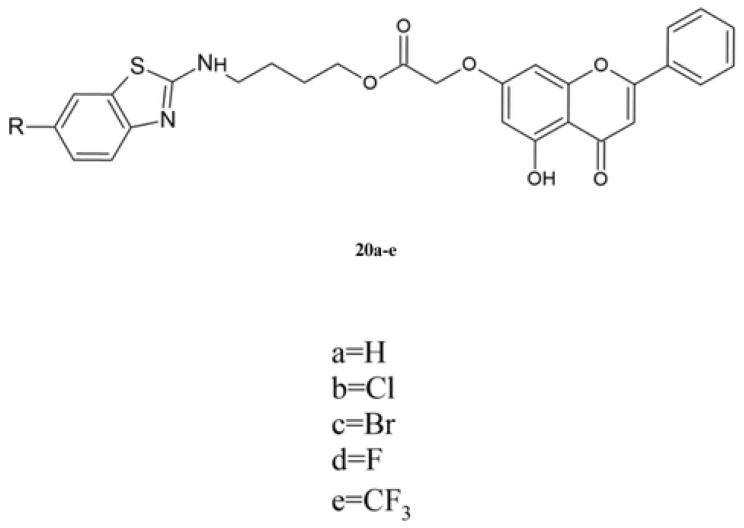
The chemical structure of derivatives **20a**–**e**.

Mistry et al. synthesized the benzothiazole derivatives of chrysin **20a**–**e** (Figure 8). They determined antioxidant activity using the DPPH method and anticancer activity against a panel of human cancer cell lines. As a reference substance, ascorbic acid and taxol were used (Table 13) [124].

As seen in Table 13, derivatives **20a**–**e** exhibit lower antioxidant activity than the reference substance. The introduction of an R group increases the activity, and the order is as follows: **20b** > **20c** > **20e** > **20d** > **20a**. Comparing the antioxidant effect of halogen derivatives **20b**–**d** shows that the highest value of IC_50_ was obtained from compounds with chloride atoms.

Compounds **20a**–**e** show high anticancer activity against the HeLa cell line, and the IC_50_ values are more than 2-times higher than those obtained for taxol (Table 13). The highest activity is shown for derivative **20a**, which does not contain an additional R group. The introduction of a halogen atom reduces the activity. However, replacement of the halogen atom by a trifluoromethyl group slightly increases the activity. Compounds **20a**–**e** show low activity against the CaSki cell line. For this line, the introduction of an R group to the benzothiazole moiety increases anticancer activity [124].

The toxicity of compounds was determined using normal cell line MDCK. The results show that derivatives **20a**–**e** exhibited low toxicity against this cell line [124].

Porphyrin shows high anticancer activity, and it is used in photodynamic therapy for cancer treatment. Developing derivatives of porphyrin holds great promise for enhancing its therapeutic potential. Such modifications can expand the range of its applications, improve efficacy and potency, and reduce adverse effects. Therefore, the pursuit of novel porphyrin derivatives represents a crucial step toward optimizing its clinical utility and addressing current limitations in cancer therapy [118,125,126,127,128,129,130].

**Figure 9:**
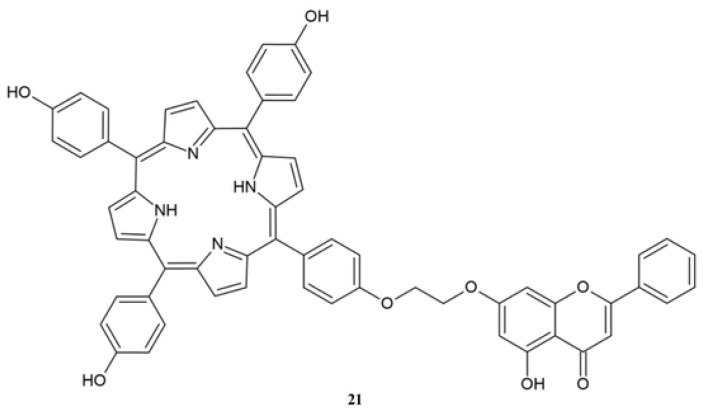
The porphyrin derivative of chrysin **21**.

Liu et al. [131] synthesized a hybrid of porphyrin derivative with chrysin **21**, which showed a high anticancer effect in in vitro tests (Figure 9).

Derivative **21** was tested against MGC-803 and HeLa cell lines both in dark and in light conditions due to different light-dependent properties of porphyrin (Table 14). The results show that anticancer activity against both HeLa and MGC-803 is similar in dark conditions. Meanwhile, in light conditions, compound **21** exhibits higher activity against HeLa cells than against MGC-803 cells. The anticancer activity of **21** against HeLa cells in light conditions was over 5-times higher compared to the activity against MGC-803 cells (Table 14) [131].

**Figure 10:**
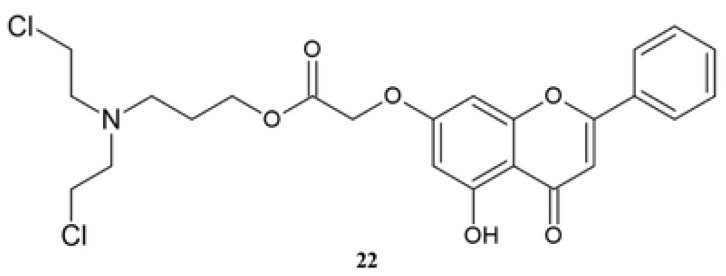
The chemical structure of derivative **22**.

Chrysin nitrogen mustard derivative **22** is synthesized via substitution reaction by adding a nitrogen mustard moiety into the flavonoid core (Figure 10) [132].

The antiproliferative activity of **22** was tested against seven human cell lines, HeLa, PC-3, DU145, MCF-7, SH-SY5Y, HepG2, and A549, and the obtained IC_50_ values are in the range of 1.43 μM to 7.86 μM, and the activity was higher against all tested cell lines than the reference substances, melphalan and chrysin **1** (Table 15).

Compound **22** shows the highest activity against HeLa, PC-3, and DU145 cell lines, and the IC_50_ is 4-times lower than the value obtained for melphalan. The activity depends on the linker between the nitrogen mustard group and the flavonoid scaffold. The analysis of the structure–activity relationship shows that compounds with a three-carbon linker exhibit higher activity than those with two-carbon or four-carbon linkers. The biological mechanism of action is based on the inhibition of the cell cycle in the G2/M phase [132].

## 4. Conclusions

Chrysin, as a natural flavone, is characterized by a broad spectrum of biological activity and low toxicity. However, its use in treatment is limited due to low bioavailability. In recent years, interest in its anticancer properties has been observed. In this review, the anticancer potential of compounds with a substituent at the C7 position of the flavone moiety and a few 5,7-disubstitute derivatives of chrysin is presented. According to this review, in most cases, the anticancer activity depends on the type of substituent. In many cases, the synthetic compounds show low toxicity. For this reason, further research in all areas of presented anticancer activity of chrysin compounds is still needed to design novel, more effective, and low-toxicity structures. In recent years, there has been a trend of chrysin moieties being connected with other compounds, which exhibited higher anticancer activity with a linker. This strategy allows the acquisition of new compounds that exhibit a broad spectrum of activity against different cancer cell lines. Moreover, derivatives of chrysin show synergistic action with used anticancer drugs. The discovery of novel modifications of flavone moieties at the C7 or/and C5 positions may lead to interesting medical applications in the future.

## Figures and Tables

**Table 1:** Anticancer activity of compound **2 [86]**.

Cell Line	IC_50_ [μg/mL]
SW480	6.3
HCT116	8.4

**Table 2:** Anticancer activity of **3**, EGGC, and its mixture [88].

IC_50_ [μM].	3	EGGC	3 + EGGC
U266	16.47	49.15	5.228

**Table 3:** Anticancer activity of **4**–**5 [89]**.

IC_50_ [μM]	Compound		
	1	4	5
SGC-7901	5.8	2.2	2.6
HT-29	3.1	2.5	1.9

**Table 4:** The anticancer activity of compounds **6** and **7** [99].

IC_50_ [μM]	Compound	
	6	7
HCT116	1.99	1.56
MOLT-4	7.05	8.69
K562	7.05	8.69
HepG2	17.53	4.93
MCF-7	17.6	26.18
A549	20.01	33.13
Caco-2	5.89	4.83
Mero-14	7.65	8.71

**Table 5:** The anticancer activity of compound **8** [100].

IC_50_ [μM]	Compound 8
MGC-803	23.83
MFC	27.34
MCF-7	40.47
HepG2	35.73

**Table 6:** The anticancer activity of compound **9** [101].

Inhibition of Growth [%]	Compound 9
K562	5
A549	2
HEL	27
PC3	0

**Table 7:** The anticancer activity of compound **11a**–**f** [118,119].

IC_50_ [μM]	Compound						
	11a	11b	11c	11d	11e	11f	Doxorubicin
MDA-MB-231	3.3	2.6	6.1	5.5	9.4	6.0	2.3
MCF-7	4.2	8.4	8.6	11.7	-	-	2.9

**Table 8:** The anticancer activity of compound **12**–**13** [55].

IC_50_ [μM]	Compound	
	12	13
A549	1.71	25.89
HCT116	1.83	12.96
U251	2.01	22.78
OVCAR-3	2.12	0.37
HT-29	6.48	3.58
HCT-15	3.28	0.06

**Table 9:** The anticancer activity of compound **14** [120].

IC_50_ [μM]	Compound	
	1	14
MDA-MB-231	40.01	4.52
MDA-MB-453	28.96	8.35
MDA-MB-468	36.02	7.88
BT-549	33.97	10.17
MCF-7	32.08	25.71

**Table 10:** Anticancer activity of **15a–d [32]**.

Compound	IC_50_ [μg/mL]			
	HeLa	CaSki	SK-OV-3	MDCK
**15a**	5.643	4.872	15.213	323.1
**15b**	6.361	4.650	36.321	371.1
**15c**	5.098	8.119	12.876	246.1
**15d**	7.770	7.628	14.213	286.3

**Table 11:** Anticancer activity of **16a**–**f [121]**.

Compound	IC_50_ [μg/mL]				
	SK-OV-3	HeLa	A-549	HT-29	MDCK
**16a**	13.05	7.33	29.18	47.73	296.8
**16b**	30.19	5.02	25.81	36.21	225.7
**16c**	12.67	4.67	27.63	31.34	168.4
**16d**	34.67	40.18	25.44	21.42	163.6
**16e**	25.83	30.99	25.89	22.06	285.4
**16f**	32.17	44.57	24.21	28.37	248.5
**Gefitinib**	12.31	17.92	13.75	23.6	-

**Table 12:** The activity of compounds **17**–**18** [122].

Compound	HT-29 IC_50_ [µg/mL]	EGFR IC_50_ [µM]
**17**	8.7	0.048
**18**	4.2	0.035

**Table 13:** The activity of compounds **20a**–**e** [124].

Compound	IC_50_ [μg/mL]			
	DPPH	HeLa	CaSki	MDCK
**20a**	26.45	4.754	16.643	347.5
**20b**	13.16	5.954	12.426	337.6
**20c**	16.09	6.564	12.425	289.1
**20d**	25.44	8.124	13.156	298.1
**20e**	16.27	7.842	11.207	278.3
**Ascorbic acid**	12.72	-	-	-
**Taxol**	-	16.48	2.48	-

**Table 14:** The activity of compound **21** [131].

IC_50_ [μM]	Compound 21
HeLa (light)	26.51
HeLa (dark)	142.7
MGC-803 (light)	70.41
MGC-803 (dark)	135.2

**Table 15:** The activity of compound **22** [132].

IC_50_ [μM]	Compound		
	22	1	Melphalan
HeLa	1.43	>100	10.72
PC-3	2.32	>100	19.81
DU145	2.91	>100	15.66
MCF-7	4.90	95.31	20.21
SH-SY5Y	7.86	49.18	20.31
HepG2	11.31	>100	13.36
A549	7.34	>100	23.39
